# Performance of the efferent limb of a rapid response system: an observational study of medical emergency team calls

**DOI:** 10.1186/s13049-015-0153-8

**Published:** 2015-09-17

**Authors:** Emilie M. Sørensen, John Asger Petersen

**Affiliations:** Department of Anesthesiology and Intensive Care, Bispebjerg University Hospital, Bispebjerg Bakke 23, 2400 Copenhagen, NV Denmark

## Abstract

**Aim:**

To determine the distribution of outcomes following a medical emergency team (MET) call using a modified version of the multidisciplinary audit and evaluation of outcomes of rapid response (MAELOR) tool, and to evaluate its usefulness in monitoring the performance of the efferent limb of the rapid response system (RRS) at our institution.

**Method:**

An observational study of prospectively collected data including all MET calls at our institution during the 36 weeks study period (23 December 2013 – 31 august 2014). Outcomes of MET calls were registered 24 h after the call occurred and categorized according to the MAELOR tool.

**Results:**

Fifty-five of a total of 308 MET calls were excluded due to prior limitations in treatment. Of the remaining cases 66 % had positive outcomes. Thirty two percent of the calls resulted in transfer to the ICU, of these 73 % occurred within 4 h. Patients remained on the ward in 53 % of the cases, and 56 % of these were no longer triggering at follow up. Nine patients had died at follow-up, three without a DNAR order. Three patients were lost to follow-up, two patients were discharged from the hospital and 25 remained alive on the ward with a DNAR as a consequence of the MET call.

**Conclusions:**

ICU transfer was implemented rapidly in most cases once the decision was made, but a disturbingly large number of patients, who remained on the ward were still triggering at 24 h follow-up. We found the MAELOR-tool useful to evaluate RRS efferent limb performance.

## Introduction

It is well documented that serious adverse events (SAEs) such as cardiac arrest, unanticipated intensive care unit (ICU) admission and unexpected death among hospitalized patients frequently are preceded by signs of physiological instability [1, 2]. To prevent these events from occurring an increasing number of hospitals use rapid response systems (RRS) to identify at-risk patients early and intervene appropriately. RRS consist of an afferent limb with a track-and-trigger system based on vital signs to identify deteriorating patients early and trigger a call to the efferent limb, usually a medical emergency team (MET). MET is manned with clinicians and/or nurses with special knowledge and skills in critical care or emergency medicine [1].

Despite its widespread use unequivocal documentation of effect of RRS is lacking. A number of observational studies and and two randomized controlled trials have shown conflicting results of RRS’ effects on the overall hospital mortality or incidence of SAEs [3–6]. Care of deteriorating on the ward patients is a complex process involving a wide number of staff with different backgrounds. In order for the system to be successful individuals involved have to fulfil their specific role at each level of the system. Lack of effect of RRS’s is often blamed on suboptimal implementation rather than inherent flaws of the system and accordingly a number of studies have shown that especially failure of the afferent limb is prevalent whereas performance of the efferent limb is less well investigated [7–10]. Afferent limb failure can be fairly easily described as delayed or absent activation of the MET, however efferent limb failure is much harder to evaluate, partly due to lack of definition of clinical deterioration. The usual outcome parameters based on the abovementioned SAEs all have their drawbacks. Cardiac arrest and unexpected death are variably defined in different studies, and are highly influenced by the number of limitations in treatment issued by the MET including do not attempt resuscitation (DNAR) orders [10]. Admission to the ICU is not necessarily a bad outcome, and is also highly dependent on institutional practices. In order to overcome some of these shortcomings Morris et al recently introduced the *multidisciplinary audit and evaluation of outcomes of rapid response* (MAELOR) tool to evaluate the performance of the efferent limb of the RRS [11]. The tool has been tested in two facilities in the UK to demonstrate the feasibility of measuring the effect of RRS on patient outcomes. It was found that the tool served well to classify patient outcomes following RRS activation, to improve areas of suboptimal performance, and to operate as a benchmarking tool.

In this study we used a modified version of the MAELOR-tool. The purpose was to determine whether or not the tool was reproducible and applicable at our institution. Furthermore we examined the distribution of positive and negative outcomes, and evaluated the usefulness of MAELOR in monitoring the performance of the efferent limb of our RRS.

## Materials and method

### Study design and setting

This was an observational study of prospectively collected data on outcomes of medical emergency team (MET) calls at our institution during a 36 weeks study period (23 December 2013 – 31 August 2014). We included data on all MET calls reported to our database by the members of the MET. The study was conducted at Bispebjerg University Hospital, Copenhagen, Denmark an inner-city hospital that serves a population of 300.000 with 280 medical and 195 surgical beds, and a mixed ICU with ten beds. The manuscript was prepared in accordance with the STROBE guidelines on reporting observational cohort studies that includes but is not limited to a detailed description of: study design and settings, data collection, eligibility and exclusion criteria for participants, methods of follow up, definition of relevant variables of outcomes, and statistical methods used [12].

### MET and early warning score system

The hospital has had a fully implemented rapid response system (RRS) since 2008 that is available 24 h 7 days a week, and includes a MET staffed with a specialist in anesthesiology and an intensive care unit nurse. MET serves all departments of the hospital except for the emergency department (ED), operating room (OR), postoperative recovery area, and intensive care unit (ICU). Patients are eligible for MET review regardless of preexisting limitations in treatment. In May 2012 the single parameter track and trigger system at our hospital was replaced by an aggregated weighted track and trigger system (AWTT) based on NEWS that includes measures for respiratory rate, arterial hemoglobin oxygen saturation, pulse rate, systolic blood pressure, level of consciousness according to AVPU score, temperature, and whether the patient receives supplementary oxygen (Table 1) [13]. Nurses assign scores and each vital sign can be assigned between 0 to 3 points (supplementary oxygen 0 or 2) depending on how much it deviates from a predefined threshold; the values are added to an aggregated score from 0 to 20, higher scores indicating more severe disease. An escalation protocol that directs the type of clinical response and competency of the provider according to early warning score (EWS) triggers is an integrated part of the system. According to this protocol MET review is advised at EWS ≥ 7, and immediate review by a senior physician or MET is mandatory for patients with EWS ≥ 9. Implementation of EWS at our institution was conducted through involvement of specially trained members of the nursing staff and physicians together with heads of departments. All new employees are introduced to the system and there is ongoing training for all healthcare providers on general wards in assessment and initial stabilization of acutely deteriorating patients and collaboration with MET.

**Table 1:** Early warning score with physiological parameters and corresponding weighted score and normal range

Vital sign	3	2	1	0	1	2	3
Respiratory Rate per min	<9		9 – 11	12 – 20		21 – 24	>24
Oxygen saturation	<92 %	92 – 93 %	94 – 95 %	>95 %			
Supplemental oxygen		YES		NO			
Temperature degrees centigrade	<35.1		35.1 – 36.0	36.1 – 38.0	38.1 – 39.0	>39	
Systolic blood pressure mmHg	<91	91 – 100	101 – 110	111 – 219			>219
Heart rate per min	<41		41 – 50	51 – 90	91 – 110	111 – 130	>130
Level of consciousness				A			V, P, U

### Data collection

Since 1 November 2013 data from MET calls have been registered electronically. Data is collected by a member of the team and includes: date of admission, date of call-out, arrival time of MET, last EWS prior to MET review, patient status after MET visit, and treatment limitations either prior to MET call or as a consequence of the call. ICU admissions and medical records including EWS were extracted from separate registers (CIS, Daintel, Denmark) and KISO (CSC, Denmark), respectively.

### Participants

All patients that received a MET call during the study period were eligible for inclusion. Patients with preexisting treatment limitations were excluded from the analysis, because the effect of MET review was considered limited in this population. Cardiac arrests were also excluded, as they were treated by a separate team. Patients were categorized as either surgical or medical, and we included ambulatory and psychiatric patients in the latter.

### Patient follow-up and outcomes

We registered clinical outcomes for all calls included during the study period 24 h after MET review and categorized them according to a modified version of the MAELOR-tool [11]. MAELOR supplies a matrix with mutually exclusive patient-related clinical outcomes that can be categorized as either positive or negative according to specific criteria. These include: admission to an ICU, alive on ward without DNAR, dead or other outcomes. ICU admission is labeled positive if it takes place within 4 h after call-out, otherwise it is negative. The time limit is chosen because previous studies have shown higher APACHE scores for patients with delayed ICU admission [14]. Patients alive on the ward at 24 h follow-up were categorized as positive outcomes if they no longer triggered, i.e. had an EWS < 7, and negative outcomes if they had an EWS ≥ 7 unless the trigger was due to new pathology or chronically impaired vital signs due to and underlying chronic comorbidity. Option three included patients not admitted to the ICU and who had died at follow-up. They were categorized as positive outcomes if death occurred in extension to treatment limitations ordered by MET; all other deaths or cardiac arrests were labeled negative outcomes. The fourth and final option includes the following positive outcomes: alive on ward with DNAR order, alive on ward with EWS ≥ 7 due to chronic condition or new trigger, and patients discharged from hospital. Patients lost to follow up were categorized as negative outcome.

### Statistical analysis

We used median and interquartile range for continuous data in descriptive statistics. Categorical variables were compared using Chi square test. Calculations were performed with RStudio, Version 0.98.501 software package (RStudio, Inc).

### Ethics

According to Danish law approval of the ethics committee is not required for observational studies, and the study was approved by the Danish Data Protection Agency (J.nr. 2013-41-1944). Data were collected and analyzed as part of a quality insurance initiative.

## Results

We collected data on 308 MET calls during the study period between 23 December 2013 and 31 August 31 2014, of which 55 were excluded due to prior limitations in treatment. The remaining 253 calls were distributed among 206 patients (53 % male) with mean age of 71 (60 – 80) years (Table 2). A total of 34 patients had two or more calls during the study period, twelve of which were within 24 h of a previous call. The majority of calls were made to patients on medical wards (67 %) including four calls to ambulatory patients and three calls to patients on the psychiatric ward. The distribution of calls throughout the day is shown in Table 3 together with distribution according to gender, day of week and ward type.

**Table 2:** Demographic data

	Female	Male	Total
Age (+/- IQR)	73 (61 – 84)	69 (58 – 78)	71 (60 – 80)
Number	97	109	206

**Table 3:** Correlation between type of ward, gender, weekday, time of day and age with outcome

	Positive outcome	Negative outcome	*p*
Surgical	54	30	0.6835
Medical	113	56	
Female	77	33	0.2397
Male	90	53	
Weekday	122	62	0.8709
Weekend	45	24	
Day (8:00 – 16:00)	64	30	0.5721
Evening (16:00 – 24:00)	50	23	
Night (0:00 – 8:00)	53	33	
>65 years	108	54	0.7679
≤65 years	59	32	

Outcomes are shown in Table 4. Positive outcomes were registered in 66 % of all cases. Eighty-one calls resulted in transfer to the ICU of which 73 % were categorized as positive. In 133 cases the patient remained on the ward, and was alive at follow up, of these patients 56 % were no longer triggering. Nine patients had died at follow-up, three without a DNAR order. Three patients were lost to follow-up, two patients were discharged from the hospital and 25 remained alive on the ward with a DNAR as a consequence of the MET call.

**Table 4:** Distribution of outcomes of the 253 included call-outs

	Positive outcome			Negative outcome			Total	
		N	(%)		N	(%)	N	(%)
Admission to ICU	1. Timely admission (<4 hrs)	59	23 %	2. Delayed admission (≥4 hrs)	22	9 %	81	32 %
Alive on ward	3. No longer triggering	75	30 %	4. Still triggering	58	23 %	133	53 %
Died	5. With DNAR	6	2 %	6. No DNAR/ cardiac arrest	3	1 %	9	4 %
Other	7. Alive with DNAR	25	10 %	9. Lost to follow up	3	1 %	28	11 %
	8. Others^a^	2	1 %				2	1 %
Total		167	66 %		86	34 %	253	100 %

^a^Patients were discharged to different hospital within 4 h

We found no statistically significant differences for outcomes between ward type, gender, day of the week, time of day, or age.

## Discussion

In this study of MET calls we used a modified version of the newly introduced MAELOR-tool to assign patient outcomes to four mutually exclusive categories, and evaluated the performance according to predefined standards for each category as positive or negative [11]. Two-thirds of all MET calls at our institution could be labeled as positive outcomes according to this tool. The largest group of poor outcomes is found among patients on the ward who still trigger 24 h after the call. They make up a concerning 23 % of all calls. Almost one-third of all MET calls result in transfer to the ICU, 73 % within the chosen time frame of less than 4 h, which still leaves a substantial number of MET-patients exposed to the risk of delayed admission. Thirty-one calls (12 %) resulted in limitations of treatment, with 20 % of these patients dead within 24 h.

MAELOR provides a useful tool to evaluate the performance of the efferent limb of RRS, both over time at the same institution and between institutions. Morris et al reported on the performance of RRS using MAELOR in a district general hospital with limited RRS coverage and a university hospital with a fully implemented 24 h RRS in place, and found a substantial difference in positive outcome rates of 75 % and 93 % between them. Transfer to the ICU was found to be delayed in 62 % and 2 % of all ICU admission respectively, and 25 % and 6 % of patients who remained on the ward were still triggering at 24 h follow-up. To be applicable for benchmarking it is mandatory that trigger tools, MET composition and case-mix are comparable between hospitals; consequently direct comparison of outcomes with our study is difficult. In addition we included all MET calls during the study period, unlike Morris who only included the first call per patient, and we used a trigger of EWS ≥ 7 in contrast to a modified EWS (MEWS) ≥ 3. Despite these differences we found an overall lower number of positive outcomes (66 %) compared to the other hospitals, and a 73 % rate of timely ICU transfers, which places our hospital between the other two. In 23 % of all calls at our institution the patients remained on the ward still triggering, compared to 15 % and 4 % of patients previously reported. So while it seems that ICU transfer is implemented fairly rapidly once the decision is made, far too many patients remain on the wards with inadequate care, many of whom might have benefited from transfer to higher levels of care in order to receive proper treatment for the underlying causes of their distress. A certain delay in ICU transfer can be acceptable if appropriate treatment is commenced on the ward in collaboration with MET, yet missed ICU transfers are to be avoided, both to ensure the best interest of the patient at risk, but also because proper monitoring and treatment of these patients often require resources both in competency and staffing, which are seldom met by general wards and could compromise care of the remaining patients. The reason so many patients stay on the ward with high triggers at our hospital is highly speculative, but could include commonly known barriers to MET activation, such as: negative feedback from MET, reluctance to call due to fear of reprimands from peers and superiors, or a feeling that a new MET call was not an option, since it already had been tried [7, 15].

There are a number of weaknesses in our study. Firstly, not all patients meeting the calling criteria are seen by MET. If MET is not activated by the staff, or if the patients are treated successfully by a doctor on the ward they will not be included in the study. We have earlier shown that many of the serious adverse events occurring at our institution are preceded by missed or delayed activation of the MET [4]. These patients could affect the results in the outcome matrix, but it is impossible to predict in which direction. Secondly, for an unknown number of patients that are transferred to the ICU directly in connection with MET call, registration to the database is lacking due to time constraints of the staff, since the nurses and physicians manning MET also take care of the patient on admission. These patients would have improved the proportion of positive outcomes in category one. Thirdly, the study period covered a rather short period of time (36 weeks); this precludes us from drawing conclusions on seasonal variations, which could stem from differences in staffing during summer or higher disease burden during winter. Finally, an inherent drawback of the MAELOR tool is that it does not describe the causes of poor outcomes; it is also quite labor intensive which limits its usefulness for continuous quality surveillance. Despite these limitations we find MAELOR to be a useful tool to monitor the performance of the efferent limb of the RRS, and it could have a role as an intermittent measure of performance at the same institution over time. However it needs further evaluation in different institutions and healthcare systems, before its general use can be recommended.

## Conclusion

In this study we evaluated the performance of the efferent limb of the RRS at our institution by using the recently introduced MAELOR tool that categorizes MET calls into clinically relevant, patient centered outcomes. We found an overall of 66 % positive outcomes according to this tool, including rapid transfer to the ICU in 73 % of all admissions to the ICU following review by the MET. However a disturbingly large number of patients (23 %) remained on the ward and were still triggering at 24 h follow-up. We found the MAELOR-tool useful to evaluate RRS efferent limb performance.
